# Adapting Neurology Residency: A Case Study and Practical Guide for Accommodations for a Disabled Resident

**DOI:** 10.1212/NE9.0000000000200332

**Published:** 2026-06-11

**Authors:** Thomas John Pisano, Laura A. Stein

**Affiliations:** From the Department of Neurology, University of Pennsylvania, Philadelphia.

## Abstract

Neurologists with disabilities remain underrepresented in the US physician workforce despite decades of legal protections mandating accommodation. Although the Americans with Disabilities Act provides a statutory framework, graduate medical education programs frequently lack structured, operational processes to implement accommodations in residency training. In this case study, a neurology program director and resident—both physicians with spinal cord injuries—describe how they adapted a neurology residency to support equivalent training while preserving educational rigor and patient safety. Before residency began, they identified accessible clinical sites, designed equivalent rotations to preserve educational rigor, and conducted audits of key clinical spaces. When environmental or scheduling demands exceeded feasible modification, they implemented alternative solutions that ensured the resident's educational exposure remained on par with nondisabled peers. Unanticipated barriers—including security policy changes and environmental hazards—further refined a structured workflow emphasizing early engagement, environmental review, and iterative reassessment. This experience informed development of a practical, replicable framework and accompanying Practical Guide. By documenting both anticipated and unforeseen barriers, this case illustrates how neurology training can evolve from ad hoc accommodation toward proactive institutional design that aligns inclusion with educational excellence.

## Background

Historically, medical training, including residency training in neurology, developed within apprenticeship structures and fixed service demands that implicitly assumed able-bodied trainees.^1^ Technical standards in medical education have been criticized for potentially excluding otherwise qualified learners with disabilities^2^ through similar assumptions that only nondisabled individuals could perform the skills required of a physician. Although the Americans with Disabilities Act (ADA)^1^ established legal protections for individuals with disabilities, health system implementation has emphasized patient-facing accessibility rather than provider inclusion. As pathways into medical school have expanded for learners with disabilities,^3^ representation within the practicing physician workforce has not kept pace; approximately 3% of US physicians report having a disability.^4^ Within neurology specifically, 5.4% of residency applicants surveyed in 2022–2023 identified as having a disability,^5^ although underreporting is likely. These data underscore a persistent gap between educational access and long-term workforce inclusion.

The implications of this gap are particularly notable in neurology, where many patients live with chronic physical and cognitive disabilities. Clinicians with lived experience of disability may demonstrate enhanced empathy,^6^ improved communication,^7^ and deeper insight into patient experience.^8^ Neurology residency requires continuous clinical coverage, particularly for inpatient services, necessitating thoughtful preresidency planning to design equivalent breadth and rigor of training schedules for incoming residents with disabilities. Without structured planning, these demands can expose accessibility barriers that extend beyond simple ADA compliance. It is important to note that accommodations are not exceptions to fairness—they are critical mechanisms to ensure that all residents, including those with disabilities, can meet the same educational standards through equivalent pathways that account for individual needs while upholding competency.

Despite mandates requiring accommodation^9,10^ and longstanding legal scholarship addressing accommodation in professional training programs,^11^ few residency programs provide standardized operational guidance for adapting rotations, schedules, or clinical workspaces.^12-15^ Implementation is frequently reactive and individualized,^12-15^ with trainees bearing the burden of disclosure and self-advocacy,^16,17^ particularly during transitions into residency training. Program directors may express support for disability inclusion while simultaneously voicing uncertainty about how to preserve fairness, workload balance, and competency standards.^15^ As a result, accommodations often emerge through informal negotiation rather than transparent institutional design. Existing policies tend to focus on procedures for requesting official workplace accommodations rather than practical guidelines for adapting the workspace, such as through clinical schedule changes and modifications to physical workspaces, leaving learners primarily responsible for identifying and advocating for necessary changes based on their individual needs. These gaps^12-15^ and the lack of formal resources emphasize the urgent need for disability-informed processes at the institutional level.^14,15,18^

Although published case reports describe successful inclusion of trainees with disabilities,^19-22^ detailed operational accounts within neurology residency remain limited. This case study documents one institution's proactive approach to adaptation, describes how an initial planning process evolved into a structured workflow, and offers a Practical Guide (eAppendix 1) to facilitate replication, aligning with emerging calls to build durable inclusion infrastructure.^18^

## Institutional Case and Lived Experience

The University of Pennsylvania Neurology Residency Program is a large tertiary academic program with 14 residents per postgraduate year and clinical responsibilities spanning multiple hospital sites. The resident (T.J.P.), who sustained a T4 complete spinal cord injury 14 years before residency and uses a wheelchair, disclosed his disability during the application process. He retains full hand function but has no lower extremity function and limited abdominal control, which affects balance during certain clinical tasks. He disclosed his disability in his personal statement, noting that it was central to his motivation to pursue neurology.

The program director (L.A.S.), who has an incomplete cervical spinal cord injury resulting in partial upper and lower extremity weakness, had completed her own training with minimal formal accommodations and significant reliance on self-advocacy. Her experience shaped a commitment to begin adaptation planning before residency start rather than after barriers emerged.

Several months before the resident's start date, structured pre-residency meetings were held. These discussions addressed anticipated barriers, including inaccessible call rooms, stroke alert response logistics, navigation between hospital buildings, and procedural space configuration. The resident expressed concerns that inaccessible environments could delay patient care, increase cognitive load, or create perceptions of unfairness among peers. Both parties emphasized preserving educational rigor and minimizing unintended burden on co-residents.

Rotation planning followed an equivalence framework. Substituted rotations were required to maintain comparable caseload, clinical intensity, and learning objectives. Navigating less accessible locations imposes a substantial cognitive burden on top of the steep learning curve all new neurology residents face. To mitigate this, the most accessible sites were incorporated early in the schedule, allowing the resident to focus on mastering core neurology skills before tackling the additional challenges of less accessible environments. To maintain educational parity with his nondisabled co-residents, each substituted rotation provided the resident with comparable clinical exposure, similar intensity, and aligned learning objectives (e.g., a more accessible hospital site with equivalent caseloads and hours). For example, the health system's 3 clinical sites included 1 main teaching hospital and 2 smaller hospitals, but one of the smaller hospitals did not have an accessible call room, so the resident completed his night requirements at the other 2 sites. He went to all 3 hospitals for day rotations because that did not require access to the call room.

Comprehensive physical space audits were conducted across outpatient clinics, inpatient workrooms, procedure suites, and call rooms. Clinical operations leadership collaborated to prioritize necessary modifications before training began. As an example of modifications completed, in the outpatient lumbar puncture suite, the sink was made ADA-compliant and moved to be within the resident's reach, a sharps container was repositioned for easier access, and the examination table was moved to create more space for the resident's wheelchair. The examination table was also confirmed to be adjustable with controls that were accessible.

Despite planning, unforeseen challenges still arose. At one hospital, new security protocols compromised building access and severely limited the accessibility of the clinical care spaces, delaying the resident's ability to respond promptly to stroke alerts. The neurology workspace was connected to the main hospital by a nonaccessible tunnel, forcing the resident to exit the building, cross a street, and enter through the emergency department (ED), which was the closest access point. However, new security measures meant that the resident's key card did not open the ED door and, when alerted to the problem, initial requests to restore access were denied. This required the resident to circle around the entire hospital to the general employee entrance, pass through a metal detector, and traverse the entirety of the hospital interior to reach the ED, which was an unacceptable delay for accessing critical spaces. The second unanticipated issue related to this location arose because of weather-limiting access. In the winter, ice and snow rendered this exterior route nearly impossible to navigate. This, understandably, was not something the scheduler or the resident had considered, as this was not a known or anticipated route. The resident traded winter rotations with his co-residents for that year and requested that future rotations at this site avoid winter months. The resident's ethos as a disabled physician is a willingness to compromise on issues that only affect him (such as less accessible workspaces), but also an unwillingness to compromise on patient safety and quality of care. In this situation, the resident's ability to provide urgent or emergent patient care was significantly impeded, and thus, the issue was escalated until he was granted the necessary ED access.

Throughout training, scheduled check-ins allowed for iterative refinement of scheduling and environmental adjustments. It is important to note that accommodations were implemented without lowering clinical expectations or redistributing workload to peers. The resident completed training, meeting the same competency benchmarks as peers, illustrating that structured planning can align inclusion with rigor.

## Evolution of a Structured Framework

Initial planning conversations evolved into a structured workflow encompassing 5 domains: (1) early preresidency consultation; (2) environmental audits; (3) schedule optimization using rotation equivalency; (4) financial planning for modifications or supplemental staffing; and (5) scheduled iterative reassessment. The Table and Figure present this sequence, while eAppendix 1 provides a detailed Practical Guide with prompts and implementation steps.

**Table:** Lessons Learned and Structured Workflow for Disability-Inclusive Residency Planning

Domain	Lessons learned	Workflow step
Pre-residency consultation and structured engagement	Early discussions allow for a shared understanding of the learner's disability and set the stage for tailored accommodations	Initiate conversations several months before the trainee's start date to identify potential barriers, plan physical space modifications, and discuss scheduling needs
Physical space audits	Comprehensive assessments of clinical and educational areas can reveal unanticipated obstacles (e.g., narrow doorways, high counters, and inaccessible call rooms)	Evaluate workrooms, call rooms, procedure suites, and clinic spaces for accessibility; implement necessary changes—such as installing automatic door openers or repositioning equipment—before the first day
Schedule environmental optimization	Residency schedules can be rigid, but careful coordination of equivalent rotations helps maintain educational rigor without affecting co-residents	Use an equivalence model to ensure that the disabled resident's learning opportunities are preserved. Reserve moonlighter support for cases where no feasible adaptation can be made, preventing workload imbalances
Educational parity and contingency planning	Equivalent clinical exposure is essential to ensure that disabled residents meet training benchmarks while minimizing disruption to co-residents	Substitute rotations of comparable rigor, caseload, and learning objectives when accessibility limits certain sites or shifts. When no true equivalency exists (e.g., inability to work nights due to a condition exacerbated by nocturnal hours), arrange moonlighter coverage or allocate budget for temporary staffing to preserve educational parity and resident well-being
Financial support mechanisms	Physical modifications, assistive technology, or moonlighter coverage may result in additional cost. Acquiring funds can be slow and prevent accommodations from being enacted in a timely manner	Secure or establish a line-item budget that program leadership can draw from, as needed, ensuring rapid and flexible responses to accommodation requests. Use colleagues in human resources and facilities
Regular iterative adjustments and open communication	Scheduled check-ins throughout the residency allow for real-time identification of emerging barriers and refinement of existing accommodations	Conduct formal reviews after each rotation or at set intervals, maintaining a transparent dialog between the resident, program director, and any relevant institutional stakeholders and note for historical reference to assist with future process improvement
Case studies to guide practice	Sharing real-world examples—like this one—helps dispel uncertainty, encourage innovative solutions, and demonstrate that trainees with disabilities can excel when given reasonable support and accommodation	Document the accommodation process and outcomes for each disabled learner, adding to an institutional and national repository of best practices and success stories
Institutional frameworks informed by disabled professionals	Policies shaped by individuals with firsthand disability knowledge are more relevant and practical. This approach ensures that GME structures evolve to meet diverse learner needs	Involve providers and trainees with disabilities in policy development and review, promoting an institutional culture that actively embraces inclusivity

**Figure:**
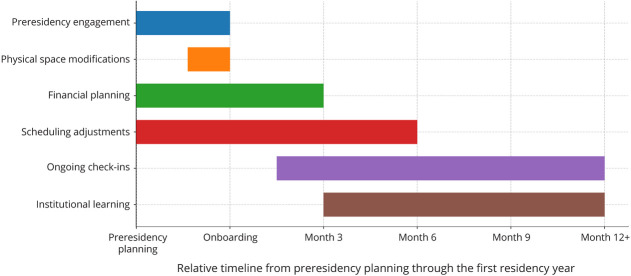
Standardized Workflow Time Line for Residency Disability Accommodations Time line illustrating the sequence of key domains for implementing accommodations for residents with disabilities over the course of a residency year. Domains include early preresidency engagement, physical space audits, scheduling and rotation adjustments, financial planning, regular iterative check-ins, and institutional learning. The workflow was initially drafted during preresidency planning discussions and refined throughout training in response to real-time challenges and unanticipated barriers. It is intended for prospective use in planning accommodations before a trainee's start date, as a monitoring tool during training to ensure that commitments are met, and for retrospective review to document and share lessons learned. The workflow is complemented by the eAppendix 1 Residency Adapting Practical Guide, which provides practical prompts, anticipatory questions, and follow-up actions for each domain to facilitate direct application in program planning and evaluation.

Drawing from our experience, we recommend that graduate medical education (GME) programs focus on 4 priorities: developing inclusive clinical and educational spaces that exceed minimum accessibility standards; establishing formal institutional guidelines in collaboration with GME leadership, disability resource professionals, and disabled health care workers; creating structured feedback mechanisms to support ongoing refinement of accommodations; and fostering cross-institutional learning to accelerate adoption of best practices. Together, these steps shift accommodation from reactive, case-by-case problem solving to proactive institutional design, strengthening workforce diversity, equivalency of training, and ultimately patient care.

Notably, financial planning is essential for future residency adaptations. Environmental modifications and occasional staffing adjustments may incur costs that exceed routine budgets. Establishing accessible funding mechanisms reduces delays and prevents accommodations from becoming dependent on informal negotiation.

Equally important was embedding reassessment into the workflow. Hospital infrastructure, security policies, and clinical demands can change unexpectedly. Formalized review points after rotations or at defined intervals allow programs to identify emerging barriers early and respond proactively.

## Implications for Neurology Training

This case reflects a transitional moment in neurology education—from individualized accommodation toward structured disability-inclusive design. Centralized processes, defined communication pathways, and dedicated funding mechanisms can replace reactive adaptations. Although developed in neurology, these principles are relevant across specialties with comparable service demands.

Sustainable inclusion requires institutional frameworks that persist beyond individual efforts. Systematic documentation of accommodation strategies can reduce uncertainty for both trainees and administrators while building a growing repository of best practices. Such transparency may also reduce stigma by normalizing disability inclusion within GME.

Disability inclusion in residency training is not solely a compliance exercise; it is a matter of educational equivalence and workforce diversification. Structured processes can help ensure that accessibility and excellence are mutually reinforcing rather than competing priorities.

## Conclusion

Decades after enactment of the ADA,^1^ residency programs often lack operational frameworks for accommodating trainees with disabilities. This case demonstrates that early planning, environmental review, rotation equivalency modeling, financial preparation, and iterative communication can preserve educational rigor while advancing inclusion.

As 2 physicians with spinal cord injuries, the authors draw from lived experience in describing this adaptation process and recognize the diversity of disability experiences and present this framework as a starting point for continued collaboration and refinement.

The structured workflow and accompanying Practical Guide provide a replicable model for shifting from reactive accommodation toward proactive institutional design. By adopting such frameworks, neurology residency programs can better allow for excellence for all learners from the first day of training.

## Glossary

ADA: Americans with Disabilities Act
ED: emergency department
GME: graduate medical education
